# Editorial: Thymus biology: development, immunodeficiency and cancer progression

**DOI:** 10.3389/fimmu.2026.1778439

**Published:** 2026-01-16

**Authors:** Yongfeng He, Yong Fan, Nicolai Stanislas van Oers

**Affiliations:** 1 Department of Cardiothoracic Surgery, Weill Cornell Medicine, New York, NY, United States; 2 Institute of Cellular Therapeutics, Allegheny Health Network, Pittsburgh, PA, United States; 3 Department of Immunology, University of Texas Southwestern Medical Center, Dallas, TX, United States

**Keywords:** combination therapy, immunotherapies, T cells, thymic epitelial tumors, thymus

The thymus is a fundamental organ of the immune system, principally responsible for the generation of functional T cells selected to recognize but not overtly respond to self-peptide-self-MHC complexes. This involves processes termed positive and negative selection processes that ensure effective antigen recognition while preventing self-reactivity (1). While most active during adolescent periods, the thymus subsequently undergoes an age-related atrophy. This organ is also highly susceptible to a range of pathophysiological alterations, including hyperplasia, autoimmune diseases, and oncogenic transformations. Among these conditions, thymic epithelial tumors (TETs) represent a rare and heterogeneous group of malignancies that derived from thymic epithelial cells. Given these dynamic changes and their profound implications for immune homeostasis and disease outcomes, continued investigation into thymic biology and pathology is essential. This Research Topic aims to explore the biological mechanisms underlying the thymus growth, regeneration and tumorigenesis, as well as to evaluate efficacy of novel anti-tumor therapies. Through a Research Topic of six manuscripts, insights into key biological and clinical questions are provided. This includes principles governing thymic selection, strategies to enhance thymic function in aged individuals, updated classification of thymic epithelial tumors and clinical outcomes for immune-based or combination therapies.

To explore the fundamental rules underlying thymic selection, Luppov et al. analyzed how a diverse set of physicochemical and sequence features of a TCR can influence the likelihood of passing thymic selection. They identified differences in selection probabilities based on CDR3 loop length, hydrophobicity and residue sizes and demonstrated how TCR sequence composition affects lineage commitment during thymic selection. To enhance the function of thymus, Zhao et al. developed a recombinant FOXN1 (rFOXN1) fusion protein. FOXN1 is a critical regulator for thymic development and the mutations in this gene can cause selective thymic hypoplasia (2). As demonstrated in this Research Topic, intravenous administration of the rFOXN1 fusion protein increases the number of thymic epithelial cells and enhances T cell generation in the thymus in the aged mice. Other strategies to improve thymic function have been previously reported. For example, subcutaneous injection of recombinant human KGF increased thymopoiesis in aged mice (3). iPSC-derived thymus organoids can support the *de novo* generation of a diverse population of functional human T cells (4).

TETs are typically classified into thymic carcinoma and thymomas. The latter can be further divided into types A, AB, B1, B2, B3 as well as metaplastic and micronodular variants. In this Research Topic, Barone and Zhang reviewed the subtypes of thymic neuroendocrine neoplasms (tNENs), which are a rare category of thymic tumors for which clinical and pathological data remain limited in the literature. They described the major subtypes of tNENs, including typical carcinoid, atypical carcinoid, large cell neuroendocrine carcinoma and small cell carcinoma, and provided representative histological images for each subtype. In this Research Topic, we also collected three articles related to immunotherapy. Immune checkpoint inhibitors (ICIs) have shown promising efficacy in both thymic carcinomas and thymomas (5–8). PDL1 expression and related pathways have been shown to be a predictor for the responsiveness of TET patients to immunotherapy (9). Shen et al. demonstrated that MRI-sequence based radiomics signature with four distinguishing features could differentiate between PD-L1 positive and negative patients with TETs and may serve as a valuable predictive tool. Luciano et al. reported that a patient with recurrent TETs achieved complete response after two cycles of Nivolumab. It is noteworthy that the patient had developed a severe immune-related myocarditis, which was successfully resolved. Interestingly, the primary tumor was histologically diagnosed as type B2/B3 thymomas in 2002, however, a pararectal lesion detected 14 years later was diagnosed as metastatic thymic carcinoma. In the literature, histological evaluation combined with longitudinal genomic analysis has been used to determine histologic transformation during disease recurrence (10). However, in this study, genetic analysis could not be performed and therefore the thymic origin of the pararectal tumor was inferred based on positive staining of Glut-1. Zhang et al. reported a series of cases in which patients received immunotherapy in combination with chemotherapy. In this cohort, five of eight patients achieved partial responses and only grade 1–2 immune-related adverse events were observed. Although the cohort size is small and monotherapy arms were not included, this study represents the first report showing the efficacy of tislelizumab in combination with chemotherapy in advanced TETs. Interestingly, the treatment efficacy was not associated with PD-L1 expression in this patient cohort. Future studies incorporating comprehensive genomic analysis together with monotherapy comparison arms may help to clarify this observation. Nevertheless, this study suggested that combination of tislelizumab with chemotherapy may serve as a promising approach to treat advanced TETs.

Overall, the articles in this Research Topic provide new insights into the biological and clinical questions regarding the thymus and thymic epithelial tumors.
